# Does Regular Physical Activity Improve Personal Income? Empirical Evidence from China

**DOI:** 10.3390/nu14173522

**Published:** 2022-08-26

**Authors:** Xinlan Xiao, Youping Yu, Qiang He, Dingde Xu, Yanbin Qi, Li Ma, Xin Deng

**Affiliations:** 1 College of Economics, Sichuan Agricultural University, Chengdu 611130, China; 2 College of Management, Sichuan Agricultural University, Chengdu 611130, China; 3 School of Public Affairs, Chongqing University, Chongqing 400044, China

**Keywords:** exercise habit, diet, income level, endogenous switching regression model, china

## Abstract

A lack of adequate exercise threatens human health, weakening human capital accumulation. The relationship between exercise and income has become the focus of attention in health economics. In terms of reducing body weight and improving physical fitness, diet and physical exercise are intertwined and become effective ways to shape a healthy state. Based on individual-level survey data from China, this study quantified the economic returns of habitual exercise behavior by using an endogenous switching regression model (ESRM) to eliminate selection bias. The study shows that (1) participants in the group with regular exercise behavior increased their income by 3.79% compared with those not exercising regularly; (2) for the group with no regular exercise behavior, regular exercise increased their income by 13.36% compared with those not exercising regularly. Additionally, empirical evidence shows that both drinking and smoking can significantly increase individual income, despite unhealthy habits. These results suggest that the habit of regular physical activity plays a vital role in increasing individual income and improving overall national health, and the effect of individual behavior on income is affected by national culture. The outcomes are empirical evidence for the Chinese government to promote Healthy China Action and support developing countries worldwide to enable habitual exercise, stimulating a policy of exercise behavior.

## 1. Introduction

Adequate exercise and reasonable diet play important roles in human physical and mental health [1,2,3]. The state of motion is innately human [4]. Diet is the basic condition for human existence. Human ancestors lived by hunting and becoming all-around outdoor athletes to survive [5]. Although people no longer need to hunt for food in a highly convenient modern society [6], human beings have an increasing demand for a balanced diet. Therefore, adequate exercise and reasonable diet are essential for human health and well-being in modern society [7,8,9].

However, the lack of regular exercise has become a global phenomenon. For example, Guthold et al. [10], based on data from 298 school-based surveys from 146 countries, regions and territories, highlighted that 85% of girls and 78% of boys globally do not meet the current physical activity standard (at least one hour per day). According to the latest research from the World Health Organization (WHO), one in four adults (1.4 billion adults) is currently not getting enough physical activity. In addition, 199IT data (Source: http://www.199it.com/archives/1305978.html) (accessed on 15 April 2022) shows that, in 2021, Japan had the highest percentage of people exercising every week in the world at 34 percent, while the United States was 18 percent and China only 13 percent. Due to the general lack of adequate exercise in modern society, obesity [11,12], depression [13], heart disease [14], sleep disorders [15] and other unhealthy conditions are emerging in large numbers. Toft et al. [16] reported that approximately 1.5–4% of the global population suffers from complex chronic diseases of severe obesity (body mass index/BMI ≥ 40 $\mathrm{kg}/m^{2}$). Zhang et al. [17] showed that the detection rate of sub-health status among Chinese medical students is 33%. The Global Risks Report [18] has warned that the world is already facing the threat of numerous emergencies, and people need to deal with the risks through adequate exercise to avoid welfare damage.

In theory, regular physical activity can offer many beneficial rewards. As for individual physical and mental health, relevant studies showed that exercise could reduce the frequency of illness, improving depression [19] and other mental illnesses, while introducing increased benefits to health and happiness [20]. In terms of external communication, regular exercise can enhance the interaction between individuals [21], promote social contact, and relieve stress [22], which can generate social capital [23]. Studies have also found that exercise can directly affect people’s appearance and body condition, reducing individual obesity [24], which in turn will generate a psychological incentive for people to maintain the results of exercise through a healthy and low-fat diet. Interestingly, these traits can translate into higher earnings through the improved ability to work. Therefore, the relationship between physical activity and mediating factors such as health, personality, physical appearance and diet may be the key to understanding how physical exercise affects personal income level. However, few studies have quantified the impact of physical activity on individual income levels. There is, therefore, an urgent need to further estimate the economic welfare effects of regular exercise to improve the enthusiasm of individuals to participate in physical exercise.

Additionally, habitually exercising may be the result of individual self-selection. If self-selection is ignored and behavioral effects are estimated [25,26,27,28], the research outcomes could be biased. Shita et al. [29], Kosteas [30], and Zhao et al. [31] suggested that the propensity score matching (PSM) method can be used to solve the problem of sample selection bias. However, Ma and Abdulai [26] believed that both observable (such as age, gender, and education) and unobservable factors might lead to selection bias. PSM can only alleviate the selection bias caused by observable factors. Ma and Abdulai [26] and Tesfaye and Tirivayi [28] found that the endogenous switching regression (ESR) model performs better when considering the selection bias caused by both observable and unobservable factors. For example, Ma and Abdulai [26] used the ESR model to address farmers’ selection bias for cooperative membership. Zheng et al. [32] used the ESR model to solve the selection bias problem of farmers’ decision to use the Internet, and Deng et al. [33] used the ESR model to solve the problem of selection bias in outsourcing the decisions of farmers. However, few studies have appropriately considered the selection bias of regular exercise behavior. The current study uses an endogenous switching regression model to evaluate the economic welfare effects of habitual exercise after considering the selection bias caused by observable and unobservable factors.

China promotes the Healthy China Initiative, which aims to cultivate residents’ preference for exercise and a reasonable diet. Based on China Labor-force Dynamic Survey (CLDS) data from 2016 and the endogenous switching regression model (ESR), this study evaluates the quantitative impact of regular exercise habits on residents’ income. Compared with previous studies, the marginal contributions of this paper are: (1) This study constructed the conduction path and mechanism from exercise to personal income, and the empirical evidence derived from China may help to improve the overall health level of the country. (2) To explain the selection bias, this study used the ESR method to explore the quantitative impact of exercise on personal income, using various identification strategies to test the robustness of the ESR estimates, leading to more accurate conclusions. This study can provide empirical evidence for the Chinese government to promote the Healthy China Action, and empirical reference for developing countries globally to introduce policies for stimulating and supporting residents’ exercise behavior and healthy diet.

## 2. Literature Review

Insufficient physical activity is harmful. For example, a lack of exercise affects nervous system function and normal brain operations [34], resulting in slow and insensitive responses and reduced brain efficiency. In addition, it can lead to shoulder and neck pain [35], mental illness [36], depression [13] and even death [37]. Of course, the impact of exercise is a long-term time series accumulation process. Therefore, when some signs of sub-health arise, people may not directly correlate them to a lack of exercise. When people lack the sufficient understanding and motivation to exercise, they cannot actively participate in it, resulting in the adverse outcomes of insufficient exercise. Almost all relevant studies emphasize that exercise should not be ignored [38,39,40].

Participation in physical activity has considerably improved economic performance. In particular, some foreign studies showed that exercise changes the level of personal income [30,41,42,43]. For example, Lechner and Sari [44] used the data of Canada’s 1994–2008 Demographic Health Survey to deduce that exercise behavior increases an individual’s income level by 10–20%. Tovar-García [45] found that those who participate in physical exercise earn higher wages, about 6–10%, compared with sedentary people. Enhanced physical exercise intensity can increase a person’s wage in stages—about 2% in the short term and 3% in the long term. Specifically, studies by Gorry [46], Tekin and Elioz [47], Cachón-Zagalaz et al. [48], Høgsbro et al. [49], and López-Bueno et al. [50] showed that participating in physical activity could improve individuals’ income levels by improving their degree of work participation and ability to work.

**Figure 1:**
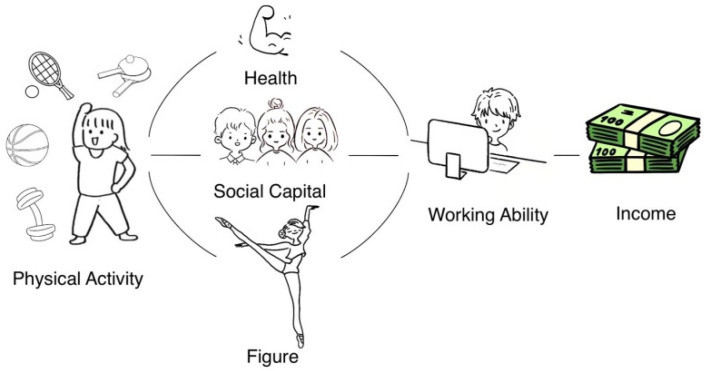
The mechanism of participating in physical exercise to improve personal income level.

Physical exercise can improve individuals’ work participation and ability by improving their physical health, body status, social capital and other factors [49,51,52], thus, improving personal productivity and personal income [50]. The specific mechanism of this is shown in Figure 1. Firstly, physical activity can directly affect the prevalence rate of individuals and improve work attendance by improving the prevalence rate. Among them, Schultz [53] indicated that individual physical quality and health status likely affect individual achievement. de Lima and Silva [52] reported that daily physical activity in boys decreased with age from 28.2% at 11–12 years to 21.2% at 16–17 years. It dropped from 19.4% to 11.1% within the same age groups for girls. Ács et al. [51] found that there is a significant correlation between regular physical activity and sick leave. On the other hand, physical exercise can reduce the prevalence and reduce the cost of disease treatment [51,54], thereby increasing personal income relatively. Secondly, physical exercise can increase individuals’ social capital, thereby indirectly improving their ability to obtain various resources in their work. Andersen et al. [21], Coalter [23], and Seippel [55] showed that physical activity helps to strengthen the connection between people (i.e., increases their social capital), inferring that information is constantly exchanged through the interpersonal relationship that is established by physical exercise. Thirdly, Lakdawalla and Philipson [24] proposed that with an increased frequency of people participating in sports, people’s physical figures and appearance would be more in line with the demand for labor force in today’s era, effectively improving the income level of workers through a “beauty premium” [56,57,58]. In addition, physical activity can increase an individual’s motivation to eat healthily [59]. In order to maintain the positive results of exercise and consolidate the good physical and body states that exercise generates, people tend to from a healthy and balanced diet structure. Therefore, physical exercise can help people obtain sufficient working capacity in all aspects, and more job opportunities, increasing their personal income.

Since individuals choose to participate in regular exercise due to self-selection, groups with higher income levels do not need to improve their working ability by participating in physical exercise. In contrast, groups who hope to improve their income level by participating in physical exercise may have a low working ability. In conclusion, selection bias may affect the estimation results due to the different initial conditions of individuals who participate in regular exercise and individuals who do not participate in physical exercise. However, existing research sparingly considers that regular exercise is a self-selection behavior and, as a result, may underestimate or overestimate the financial rewards of exercise. Therefore, this study adopted the endogenous switching regression model to eliminate selection bias, which is helpful in accurately estimating the impact of regular exercise behavior on individual income.

## 3. Data Sources, Variable Selection and Research Methods

### 3.1. Data Sources

This study used the individual-level data provided by the 2016 China Labor Dynamics Survey (CLDS2016) for an empirical analysis. CLDS2016 was a scientific sampling survey conducted by the Social Science Survey Center of Sun Yat-sen University (Guangzhou, China) in 2016, which adopted the probability sampling method that was multi-stage, multi-level and proportional to the scale of the labor force. CLDS targets the working-age population, aged 15–64, and focuses on the current situation and changes in labor force education, employment, labor rights and interests, occupational mobility, occupational protection and health, and occupational satisfaction and happiness. The data selected in this paper are highly consistent with the research topic.

The CLDS2016 sample covers 29 provinces and cities in China, with a sample size of 401 villages, 14,226 households, and 21,086 individuals, nationally representative and representative of the eastern, central and western regions. This study deleted any individual data with many missing variables and obtained 20,783 individual analysis data.

### 3.2. Variable Selection

#### 3.2.1. Dependent Variables

Referring to the study of Lechner and Downward [42], this study used individual income levels as the dependent variable. Specifically, the survey time was 2016, and the survey content was the actual situation of the working-age population in 2015. The investigation of workers’ income levels in CLDS data includes the total income of all types of workers, wage income after deducting individual income tax, social security and housing accumulation fund, wage income without deducting individual income tax, and social security and housing accumulation fund and other aspects. In practice, urban residents may have additional income, family operating income, and wage income, while rural residents may also have agricultural income. Therefore, the total income of individuals in 2015 was selected as the comprehensive measurement index of individual income level, the dependent variable in this study.

#### 3.2.2. Focus Variables

Most existing studies used exercise participation rates to measure the application of physical activity. However, this study explores the quantitative impact of individuals’ physical exercise habits on their income levels from the micro-level. Considering individual differences in exercise strategy, at the same time, this study focuses on the general effect of exercise on income, not a particular exercise behavior analysis, so this research primarily considers whether individuals exercise regularly as a measure of activity, defining focus variables as individual participation in regular exercise.

#### 3.2.3. Control Variables

According to previous studies [41,44,60], the study also controlled for characteristics such as gender, age, education level, presence of a spouse, social capital, and residence type, which are thought to influence individuals’ decision to engage in regular exercise and personal income. For example, Huang and Humphreys [61] discussed the influence of physical exercise participation on personal life satisfaction and subjective well-being by controlling individual characteristics (such as age and education). Similarly, the study of Hyytinen and Lahtonen [41] and Lechner and Sari [44] controlled for these variables. Meanwhile, Cornelißen and Pfeifer [62] reported that individual characteristics (such as age and education) would influence the behavior of participating in regular physical activity. The model variables and summary statistics are shown in Table 1.

### 3.3. Research Methods

This study investigates the quantitative relationship between physical exercise habits and personal income. Due to the fixed personal and environmental characteristics, individuals may self-select exercise instead of randomly exercising. There are also some invisible factors affecting individual decisions on whether to participate in regular physical exercise. Such selection outcomes can lead to the deviation problem, and if this selection bias is not eliminated, it can lead to inconsistent estimation results. Many scholars adopt PSM estimation techniques in practical research to correct selection bias [29,30,31]. However, the PSM method only considers observable heterogeneity and does not consider heterogeneity caused by unobservable factors. Therefore, the endogenous switching regression model (ESRM) can consider the heterogeneity caused by observable and unobservable factors to correct the sample selection and obtain unbiased and consistent estimation results.

All individuals have only two choices: participate in physical exercise regularly or not participate in regular physical exercise. When =1, individuals choose to participate in physical exercise regularly. Unfortunately, $C_{i}^{*}\;$ cannot be directly observed but expressed by the equation as follows:

(1) $$\;C_{i}^{*}=\gamma_{j}Z_{ij}+v_{i}\;,\;\mathrm{when}\;C_{i}^{*}>0,C_{i}=1;\;\;\mathrm{when}\;C_{i}^{*}<0,C_{i}=0$$

where $C_{i\;}\;$ is a binary selection variable, “$C_{i}$ = 1” indicates that the *i*th person exercises frequently, and “$C_{i}$ = 0” indicates that the *i*th person does not exercise regularly; $Z_{ij}$ represents a vector set containing some characteristic variables of an individual and the environment, such as the individual’s age, gender, and education level. γ  represents a vector set, which is $Z_{ij}$; the estimated coefficient of the vector, $v_{i}$, is an error term with zero mean and follows the standard normal distribution.

This paper aims to analyze the impact of participation in physical activity on personal income. Therefore, the following linear equation is set to express:

(2a) $$Y_{1i}=\sum_{j=1}^{n}\beta_{1j}X_{1ij}+\mu_{1i}\;\mathrm{when}\;C_{i}=1$$

(2b) $$Y_{0i}=\sum_{j=1}^{n}\beta_{0j}X_{0ij}+\mu_{0i}\;\mathrm{when}\;C_{i}=0$$

where $Y_{i}$ stands for personal income and $X_{ij}$ represents a vector set of explanatory variables, such as an individual’s age, gender, and education level. $\beta_{j}$ represents the coefficient to be estimated of the corresponding variable; $\mu_{i}$ is a random error term.

Drawing on the research of Lokshin and Sajaia [63], the factual and counterfactual expectations of individuals’ income can be estimated when they participate in physical exercise and when they do not. Specifically, factual income refers to the expected value of income of individuals participating and not participating in physical exercise, expressed as in Equation (3a,b). Counterfactual income refers to the expected value of income of people who exercise regularly if they do not participate in exercise, and those who do not exercise regularly if they participate in physical exercise, expressed as Equation (4a,b) The equations are:

(3a) $$E\left(Y_{1i}|C_{i}=1\right)=\beta_{ij}X_{1ij}+\sigma_{\mu 1v}\lambda_{1i\;}$$

(3b) $$E\left(Y_{0i}|C_{i}=0\right)=\beta_{0j}X_{0ij}+\sigma_{\mu 0v}\lambda_{0i}\;$$

(4a) $$E\left(Y_{0i}|C_{i}=1\right)=\beta_{0j}X_{1ij}+\sigma_{\mu 0v}\lambda_{1i}\;$$

(4b) $$E\left(Y_{1i}|C_{i}=0\right)=\beta_{1j}X_{0ij}+\sigma_{\mu 1v}\lambda_{0i\;}$$

Furthermore, there are differences between $Z_{j}$ and $X_{j}$ [64]. $Z_{j}$ and $X_{j}$ can overlap, but at least one variable belongs to $Z_{j}$ as an instrumental variable that does not belong to $X_{j}$. According to the peer effect theory [64,65], peer behavior is an important determinant of individual behavior. Therefore, most papers followed the peer effect theory to select instrumental variables. For example, Xu et al. [66] selected peers’ non-farm work ratio as an instrumental variable to represent households’ decisions on non-farm work. Ma et al. [64] selected peer non-farm work decisions as instrumental variables representing households’ decisions about non-farm work. Deng et al. [67] selected the average share of Internet use by households other than the households considered in the same village as an instrumental variable for Internet use. According to this concept, the instrumental variable of regular participation in physical exercise can be defined as the proportion of other individuals exercising in the same village other than the individual under consideration (*n*−1), which can be expressed as follows:

Peer’s exercise = Number/(Total − 1)(5)

Peer participation in physical exercise is the instrumental variable. The number represents those people in the same village who exercise regularly, while the total represents the total number of people in the village.

From the ESR model, the Average Treatment Effect on the Treated (ATT) for the income of individuals who regularly participate in physical exercise can be expressed as follows:

(6a) $$\mathrm{ATT}=E\left(Y_{1i}|C_{i}=1\right)-E\left(Y_{0i}|C_{i}=1\right)=\left(\beta_{1j}-\beta_{0j}\right)X_{1ij}+\left(\sigma_{\mu 1v}-\sigma_{\mu 0v}\right)\lambda_{1i}$$

The Average Treatment Effect on the Untreated (ATU) on the income of individuals who do not regularly participate in physical exercise can be expressed as follows:

(6b) $$\mathrm{ATU}=E\left(Y_{1i}|C_{i}=0\right)-E\left(Y_{0i}|C_{i}=0\right)=\left(\beta_{1j}-\beta_{0j}\right)X_{0ij}+\left(\sigma_{\mu 1v}-\sigma_{\mu 0v}\right)\lambda_{0i}$$

## 4. Results

### 4.1. Mean Difference between Regular and Non-Regular Exercising Individuals

Mean differences can help to explain data structures, providing evidence for empirical models. Table 2 summarizes the descriptive results showing the difference between individuals who exercise regularly and those who exercise infrequently. There are significant differences between regular and non-regular exercise individuals in this study. More specifically, people who exercise regularly have a lower average age than those who do not. Individuals who exercise regularly are more educated than those who do not and have more social capital.

Table 2 also lists the differences in income levels between regular and non-regular exercisers. Table 2 illustrates that individuals who exercise infrequently earn less personal income than those who exercise regularly. Thus, the descriptive results suggest that exercise habits may be the key to understanding individual income levels. The data also show some differences in most variables between those who exercise regularly and those who do not. As a result, whether an individual participates in physical activity frequently may not be random, leading to insignificant differences in income levels between people with and without exercise habits. Therefore, the ESR model solves the selection bias, accurately identifying the quantitative impact of physical activity on personal income.

### 4.2. Empirical Analysis

#### 4.2.1. Determinants of Regular Exercise and Personal Income

The estimates of the determinants of regular participation in physical activity and the impact of regular exercise on individuals are reported in Table 3. Firstly, the statistical index of $\rho_{regular}$ is significant, indicating that the decision of individuals to participate in physical exercise is not random, and there is selection bias. Secondly, the Wald test for equation independence is significant at the 1% level, so the null hypothesis (no correlation between error terms) can be rejected. The findings confirm that both observable and unobservable factors influence an individual’s decision to engage in regular exercise and its outcomes in participating in physical activity. Therefore, the ESR method is suitable for this study.

The second column of Table 3 reports the determinants of an individual’s regular physical activity participation. Among them, the higher the level of education, the higher the enthusiasm for physical exercise. The study also observed that peer influence has a positive and significant effect, suggesting that participation in physical activity can influence peers. Additionally, under the current consensus that smoking and alcohol can cause injury, smoking significantly negatively impacts physical exercise, while drinking has a significant positive impact on physical exercise. Considering marriage and employment, both significantly negatively impact participation in physical activity. 

Columns 3 and 4 of Table 3 show the effect of physical activity on the income levels of those who do or do not participate in physical activity regularly. The estimates suggest that marriage and work have a positive and significant effect on the personal income of participants and non-participants. Although marriage has a significant negative effect on participation in physical activity, it suggests that, while running a marriage, family and work may limit individuals’ participation in physical activity, there would be a more considerable increase in income for individuals who still chose to participate in regular exercise. Furthermore, empirical data suggest that both drinking and smoking increase individual income, despite unhealthy habits. While the impact of social capital on personal income is not statistically significant for those with exercise habits, for people without exercise habits, income rises by 1% for every unit of increase in social capital. 

#### 4.2.2. Estimating ATT and ATU

**Figure 2:**
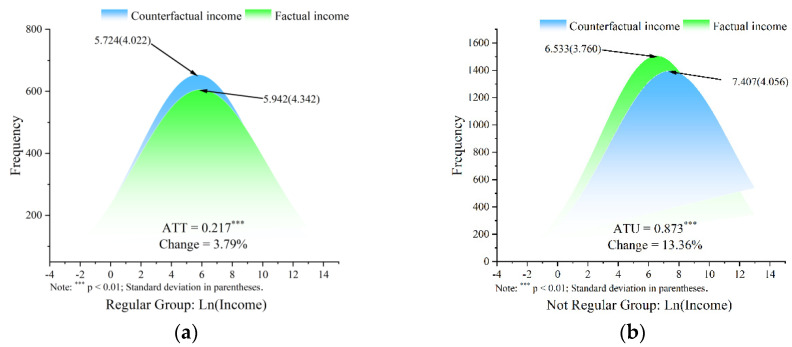
(**a**) Factual and counterfactual income of individuals with physical exercise habits; (**b**) factual and counterfactual income of individuals who do not have physical exercise habits.

Table 3 does not report the quantitative impact of regular exercise habits on personal income. Therefore, based on the estimated results of ESR, this study calculated the average treatment effect, as shown in Figure 2a,b.

In Figure 2a, the blue section represents the counterfactual income, or the income of a person with frequent exercise behavior if they do not exercise often. The green part represents the factual income or the actual income of a person with frequent exercise behavior. Figure 2a ATT represents the mean difference between factual and counterfactual income, the income effect of regular exercise behavior. The sign of ATT is positive and significantly different from zero, indicating that regular exercise behavior can significantly increase earnings. Specifically, for the group with regular exercise behavior, the state of regular exercise increased their income by 3.79% compared to those without regular exercise.

In Figure 2b, the blue part represents the counterfactual income, which is the income of a person who does not exercise regularly if they then choose to exercise regularly. The green part represents the factual income, which is the actual income when a person does not exercise regularly. Figure 2b ATU represents the mean difference between counterfactual income and factual income, which is the income effect of regular exercise behavior. The sign of ATU is positive and significantly different from zero, indicating that groups that do not exercise regularly may earn higher incomes if they exercise regularly. Specifically, for the group with no regular exercise behavior, the states with regular exercise behavior increased their income by 13.36% compared with those without regular exercise.

In summary, the data depicted in Figure 2 show that regular exercise brings higher income returns for both groups with or without regular exercise. In particular, this study found that $\rho_{regular}<0$ (in Table 3) indicates a positive selection bias, indicating that individuals with a higher-than-average income are more willing to exercise regularly. Consequently, the effect of exercise on income would be overestimated if selection bias was not taken into account. 

## 5. Discussion

Based on the 2016 China Labor Dynamics Survey data, this study uses an endogenous switching regression model (ESRM) to eliminate selection bias and quantitatively estimate the impact of regular exercise behavior on income levels.

In exploring the influencing factors of regular exercise behavior, this study found that the higher a person’s level of education, the more likely they are to choose to participate in regular exercise behavior, which is consistent with the results reported by Werneck et al. [68]. Notably, some studies show that education is not significantly related to physical exercise [69,70], perhaps due to an endogeneity problem caused by the failure to resolve confounding factors, or it may be related to the selected characteristics of different sample groups. In addition, we observed a negative effect of smoking on physical activity and a positive effect of alcohol consumption on physical activity, which is consistent with the results reported by scholars such as Yurdalan et al. [71] and Piazza-Gardner and Barry [72], possibly because smoking significantly reduces muscle strength, explosive power, endurance and coordination. Alcoholic beverages are beneficial to exercise to a certain extent, and some people choose to burn off the calories they consume by exercising, increasing their exercise rate. Cobb-Clark et al. [73] also mentioned that in order to obtain more health rewards, the individual’s internal control ability can drive them to rationally choose among regular exercise, healthy diet, moderate alcohol consumption and avoiding smoking. We also observed that marriage and work had a significant negative impact on physical activity participation, which may be related to the amount of discretionary time between the two, with married or busy working groups participating in physical activity less than those not married or with more flexible jobs.

For individual income, we found that both drinking and smoking behaviors can increase the general level, despite unhealthy habits, which is different from the results reported by Dilmaghani [74], who found that drinking behavior can increase individual income through Canadian General Social Survey data, while the effect of smoking behavior is not significant, and a wage penalty can even occur. The possible reason for this difference is that the cultural background is different. Under China’s tobacco and alcohol culture, these factors can remove the distance between people and lead them form closer social relationships, thereby increasing social capital. However, the impact of social capital on personal income is not statistically significant for people with exercise habits, which is similar to the results reported by Arsal et al. [75], who reported that social capital has a positive impact on income through trust, social network and norms. The social capital scale can help individuals form a positive attitude, but the effect is not significant; however, there is no denying that it remains economically important. For people without exercise habits, the impact of social capital on personal income was positively correlated. This finding is similar to Shangguan and Peng [76], who believe that the use of the Internet by farmers is conducive to the accumulation of social capital, which can reduce the income gap and achieve more equitable income distribution while increasing farmers’ income. Therefore, physical exercise can promote the accumulation of social capital and achieve common prosperity.

After controlling for selection bias, this study found that exercise can increase average income by 3.79%, which is lower than that reported by Tovar-García [45], Lechner [77] and Kosteas [30], who found that exercise increases average earnings by at least 5%. The reason why the quantitative research results are lower than this study may be due to the failure to choose an appropriate method to consider the problem of selection bias caused by both observable and unobservable factors, resulting in an underestimation of the impact of exercise on income levels. Therefore, it is necessary to choose appropriate methods to evaluate the economic outcomes of behavior. This study also found that the income level of participants who do not exercise regularly increases by 13.36% if they decide to exercise regularly, indicating that exercise can induce better economic effects and inspire policymakers to build more sports facilities in the community.

The strength of this study is that, when facing the problem of selection bias in the decision of individuals to participate in physical exercise, we adopt the ESR model to consider the impact of both unobservable and observable factors, and further focus on the quantitative impact of regular exercise behavior on personal income. However, due to the long period of the impact of exercise behavior on labor income level, or the existence of individual differences, it is difficult for us to test or predict in the short term, especially because different labor markets have various heterogeneity characteristics. Therefore, further research is needed to verify its application and role in the labor market.

## 6. Conclusions and Recommendations

This study examines the factors influencing individuals’ choices to participate in regular physical activity and the effect of participating in regular physical activity on individual income levels. The study used the 2016 data from the China Labor-force Dynamic Survey (CLDS), which covers 29 provinces and cities in China. A simple comparison of the personal income of participants and non-participants revealed some significant differences. The results show that sample selection bias could occur if results are estimated without considering the decision to participate in exercise. Therefore, to account for confounding factors affecting the differences, an endogenous switching regression (ESR) model was adopted, taking into account the observed and unobservable factors to address the problem of selectivity bias.

The empirical results show a significant positive relationship between regular exercise and personal income. For the group with regular exercise behavior, their income increases by 3.79% when moving from a state of no regular exercise to a state of regular exercise. For the group without regular exercise behavior, their income increases by 13.36% when moving from a state of no regular exercise to a state of regular exercise. In terms of factors influencing individuals’ decision to participate in physical activity, studies have shown that variables such as education, alcohol consumption, social capital and peers have significant and positive effects on regular participation in physical exercise. The results of this study suggest that participation in physical activity can play an essential role in increasing an individual’s income and improving the overall health of a nation.

Some policy implications can be drawn from the overall findings of this study. The region where individuals live often influences their decision to join the active population, suggesting that government policies to improve sports infrastructure would increase the number of people participating in physical activity and, thus, improve people’s health. Therefore, the government should introduce relevant policies such as “full coverage of sports infrastructure” to stimulate residents’ exercise behavior. In addition, the government could increase the support for sports and fitness activities and increase the construction of leisure and exercise spaces such as greenways to improve the current situation of low participation in sports and exercise. Simultaneously, establishing and improving food procurement, storage, processing management and other systems; using relevant policies on dietary safety and balance to effectively protect personal health; promoting healthy diets while promoting exercise; and encouraging people to consciously develop healthy eating habits will, thus, ensure the intake of nutrients and improve physical function and health. Such expenditures may encourage individuals to participate in physical activity and contribute to achieving the “Healthy China 2030” strategy’s healthy development goals.

## Figures and Tables

**Table 1:** Variable definition and descriptive statistics.

Variable	Definition	Mean	SD
Income	Respondent’s total income in 2015 (CNY)	18,304.00	23,441.73
Exercise	Does the respondent exercise regularly (1 = yes; 0 = no)	0.32	0.47
Age	Respondent’s age (years)	43.81	14.61
Education	Whether the respondent has a high school education or above (1 = yes; 0 = no)	0.30	0.46
Gender	Respondent’s gender (1 = male; 0 = female)	0.48	0.50
Marriage	Whether the respondent is married (1 = yes; 0 = no)	0.82	0.39
Job	Does the respondent have a regular job (1 = yes; 0 = no)	0.64	0.48
Cigarette	Whether the respondent has the habit of smoking (1 = yes; 0 = no)	0.27	0.44
Alcohol	Whether the respondent has a drinking habit (1 = yes; 0 = no)	0.19	0.40
Social capital	The number of respondents who maintain close contact at the survey site (number)	11.14	50.71
Rural area	Whether the respondent lives in a rural area (1 = yes; 0 = no)	0.63	0.48

**Table 2:** Difference in average characteristics between regular and not regular physical activity.

Variable	Regular	Not Regular	Diff.
Income	20,942.69 (26,249.03)	17,078.01 (21,909.06)	3864.68 ***
Age	42.32 (15.38)	44.5 (14.18)	−2.17 ***
Education	0.48 (0.50)	0.22 (0.42)	0.25 ***
Gender	0.48 (0.50)	0.47 (0.50)	0.01
Marriage	0.75 (0.43)	0.85 (0.36)	−0.10 ***
Job	0.56 (0.50)	0.67 (0.47)	−0.11 ***
Cigarette	0.25 (0.43)	0.28 (0.45)	−0.03 ***
Alcohol	0.20 (0.40)	0.19 (0.39)	0.01 **
Social capital	12.75 (55.04)	10.40 (48.55)	2.35 ***
Rural area	0.46 (0.50)	0.71 (0.45)	−0.25 ***

Note: Standard deviations are in parentheses; ** *p* < 0.05, *** *p* < 0.01.

**Table 3:** Determinants of regular exercise and determinants of personal income.

Variables		Income	
	Selection	Regular	Not Regular
Age	0.003 *** (4.257)	−0.024 *** (−8.470)	−0.035 *** (−18.194)
Education	0.380 *** (15.693)	0.274 *** (3.629)	0.393 *** (5.955)
Gender	0.120 *** (4.717)	0.382 *** (4.788)	0.712 *** (12.435)
Marriage	−0.247 *** (−8.422)	1.020 *** (10.184)	1.153 *** (15.031)
Job	−0.232 *** (−10.681)	8.429 *** (107.905)	7.581 *** (121.969)
Cigarette	−0.147 *** (−5.138)	0.212 ** (2.311)	0.309 *** (5.035)
Alcohol	0.092 *** (3.274)	0.251 *** (2.881)	0.208 *** (3.406)
Social capital	0.000 ** (2.096)	0.000 (1.349)	0.001 ** (2.175)
Rural area	−0.002 (−0.081)	−0.153 ** (−2.012)	−0.524 *** (−8.607)
Province dummies	Yes	Yes	Yes
Peer’s exercise	2.358 *** (33.477)		
Constant	−1.300 *** (−13.356)	1.994 *** (7.022)	1.958 *** (7.401)
$\sigma_{regular}$		2.543 *** (50.674)	
$\rho_{regular}$		−0.162 *** (−4.224)	
$\sigma_{not}$			2.629 *** (87.685)
$\rho_{not}$			−0.056 (−1.496)
Wald test of indep. eqns.	19.583 ***		
Log pseudolikelihood	−60,188.067		
Observation	20,783		

Note: The dependent variable of Income is log form; t-values are in parentheses; ** *p* < 0.05, *** *p* < 0.01.

## Data Availability

The datasets that were used and/or analyzed in the current study are available from the corresponding author upon reasonable request.
